# GRAF1 forms a complex with MICAL-L1 and EHD1 to cooperate in tubular recycling endosome vesiculation

**DOI:** 10.3389/fcell.2014.00022

**Published:** 2014-05-27

**Authors:** Bishuang Cai, Shuwei Xie, Steve Caplan, Naava Naslavsky

**Affiliations:** Department of Biochemistry and Molecular Biology and the Fred and Pamela Buffett Cancer Center, The University of Nebraska Medical CenterOmaha, NE, USA

**Keywords:** BAR domain, PH domain, tubulation, EHD1, MICAL-L1, GRAF1, tubular recycling endosome, vesiculation

## Abstract

The biogenesis of tubular recycling endosomes (TREs) and their subsequent vesiculation after cargo-sorting has occurred, is essential for receptor and lipid recycling to the plasma membrane. Although recent studies have implicated the C-terminal Eps15 Homology Domain (EHD) protein, EHD1, as a key regulator of TRE vesiculation, additional proteins involved in this process have been largely uncharacterized. In the present study, we identify the GTPase Regulator Associated with Focal adhesion kinase-1 (GRAF1) protein in a complex with EHD1 and the TRE hub protein, Molecules Interacting with CasL-Like1 (MICAL-L1). Over-expression of GRAF1 caused vesiculation of MICAL-L1-containing TRE, whereas GRAF1-depletion led to impaired TRE vesiculation and delayed receptor recycling. Moreover, co-addition of purified EHD1 and GRAF1 in a semi-permeabilized cell vesiculation assay produced synergistic TRE vesiculation. Overall, based on our data, we suggest that in addition to its roles in clathrin-independent endocytosis, GRAF1 synergizes with EHD1 to support TRE vesiculation.

## Introduction

Endosomes are a primary “vehicle” for protein transport within the cell (Jovic et al., 2010). This transportation includes the sorting and delivering of cargo molecules to their corresponding destinations, which depends upon a series of well-coordinated membrane fission and fusion events. A large group of cytosolic proteins assemble onto membranes to influence their shape, and facilitate the formation of a budding vesicle. These vesicles/carriers are then transported along microtubule tracks to a specific destination where they eventually fuse with a target membrane and release their cargo. Such proteins often assemble as a complex and can act as an “operational unit.”

We recently identified a protein that serves as a membrane-hub on tubular recycling endosomes (TREs) (Rahajeng et al., 2012), and coordinates multiple events that shape the membranes during endocytic transport (Giridharan et al., 2013). Molecules Interacting with CAsL-Like1 (MICAL-L1) is a novel TRE marker (Sharma et al., 2009), which recruits and stabilizes a battery of proteins that leads to a direct impact on membrane shaping (Giridharan et al., 2013). MICAL-L1 recruits many essential membrane modulators to TREs, including Rab8 (Sharma et al., 2009) and proteins that possess Bin–Amphiphysin–Rvs (BAR) domains (summarized in Figure 8A). Such BAR domain-containing proteins induce membranes into highly curved shapes (McMahon and Gallop, 2005; Zimmerberg and Kozlov, 2006) and include the N-BAR protein Amphiphysin/Bin1 (Pant et al., 2009), and the F-BAR protein Syndapin2, also known as PACSIN2 (Braun et al., 2005; Giridharan et al., 2013).

MICAL-L1 also interacts with the C-terminal Eps15 Homology Domain (EHD) proteins, EHD3 and EHD1 (Sharma et al., 2009; Kieken et al., 2010), which are involved in membrane tubulation and vesiculation, respectively (Cai et al., 2013). Also crucial for TRE biogenesis is the generation of phosphatidic acid (PA), an essential lipid component of TRE tubules that binds and recruits MICAL-L1 and Syndapin2, thus promoting F-BAR-induced tubulation (Giridharan et al., 2013). EHD1 subsequently joins this complex on TRE where it binds to both MICAL-L1 and Syndapin2 and performs scission, giving rise to newly formed recycling endosomes (Cai et al., 2013; Giridharan et al., 2013).

Along with the growing consensus for EHD1 as a vesiculator of TRE (Jakobsson et al., 2011; Cai et al., 2012, 2013), there is also evidence suggesting that additional proteins are involved in the scission process. For example, EHD1 directly interacts and cooperates with the lipid-modifier enzyme, cPLA2α, to initiate and facilitate vesiculation, thus supporting the generation of transport vesicles (Cai et al., 2012). Another protein with potential to be involved in TRE vesiculation is the GTPase Regulator Associated with Focal adhesion kinase-1 (GRAF1; also known as Oligophrenin-1-Like). GRAF1 is a protein that cooperates with other proteins to remodel tubulo-vesicular clathrin-independent carriers (CLICs) derived from the plasma membrane (Lundmark et al., 2008), thus regulating endocytosis.

**Figure 1:**
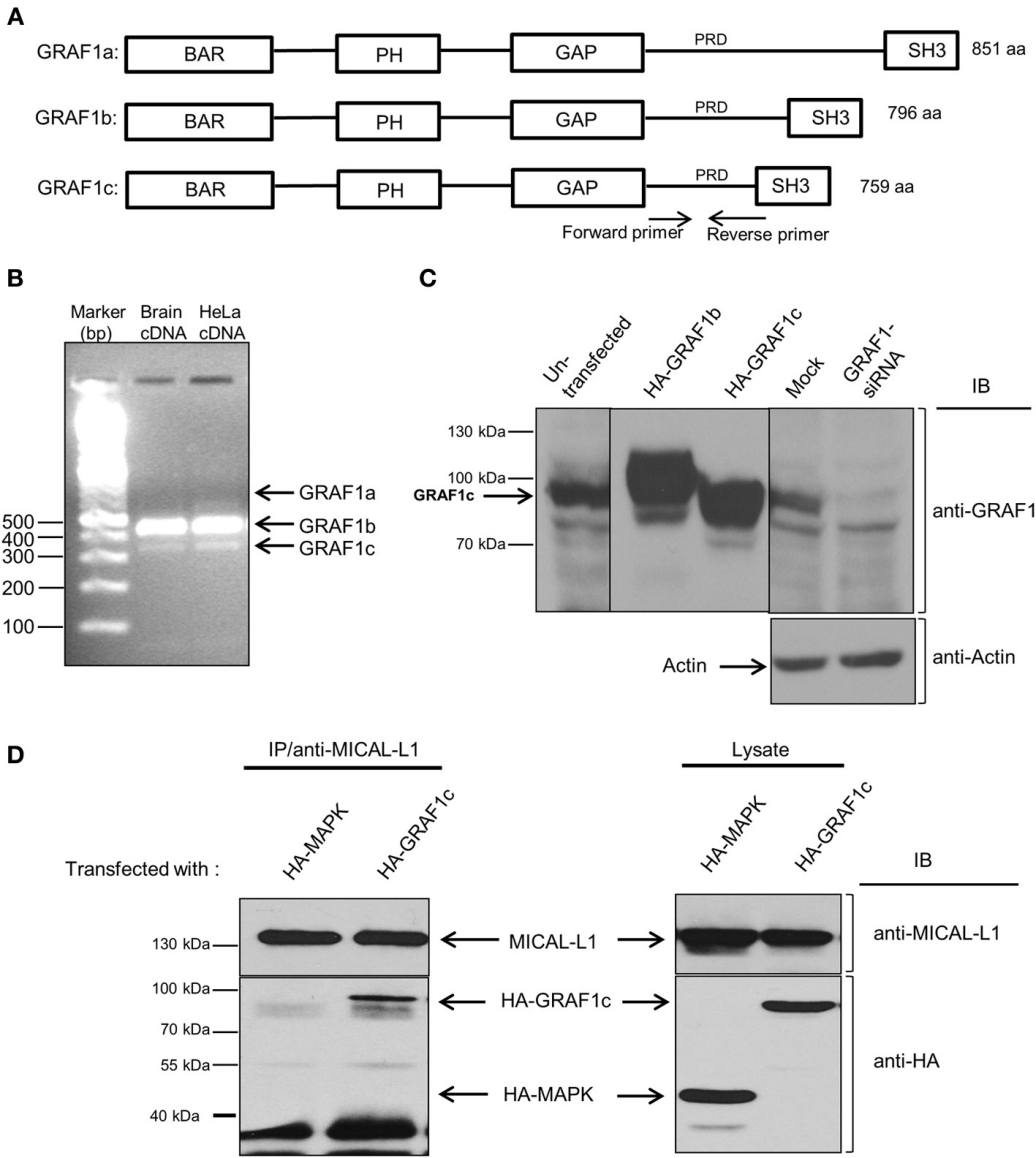
**HeLa cells predominantly express the GRAF1c isoform, which interacts with the hub-protein, MICAL-L1. (A)** The domain architecture of the three GRAF1 isoforms. **(B)** GRAF1 isoforms (a, b, and c) were amplified from either human brain or HeLa cDNA libraries with two primers as indicated in **(A)**. **(C)** Cells were either left in culture, or transfected with either HA-GRAF1b, or HA-GRAF1c for 16 h. In the left panel, the cells were subsequently mock-treated or treated with GRAF1-siRNA for 72 h before being lysed on ice. Lysates were subjected to 8% SDS-PAGE, followed by immunoblotting with anti-GRAF1 and anti-Actin antibodies (loading control). **(D)** Cells were either transfected with HA-MAPK as a negative control or HA-GRAF1c. After 48 h, cells were lysed for 1 h in buffer containing 50 mM Tris pH 7.4, 150 mM NaCl, 0.5% Triton X-100 and protease inhibitors. Cell lysates were incubated with mouse anti-MICAL-L1 antibody overnight. Protein L beads were added to the mixture of cell lysate and MICAL-L1 antibody for 3 h at 4°C. Beads were washed in buffer containing 50 mM Tris pH 7.4, 150 mM NaCl, 0.1% Triton. Proteins were eluted by adding SDS loading buffer. Samples were subjected to 8% SDS-PAGE, followed by blotting with anti-HA and anti-MICAL-L1 antibodies.

GRAF1 belongs to a family of proteins, all of which are comprised of a similar domain-configuration (see scheme in Figure 1A) consisting of: an N-terminal BAR domain, a pleckstrin homology (PH) domain, a Rho-GAP, a proline-rich domain (PRD) and a Src homology 3 (SH3) domain (Doherty and Lundmark, 2009). The PH domain is found in a variety of proteins, and it directly binds to Phosphatidylinositol-4,5-bisphosphate (PIP2) (Harlan et al., 1995). The BAR and PH domains of GRAF1 work cooperatively to bind to highly curved PIP2-containing tubular and vesicular membranes (Lundmark et al., 2008). Via its Rho-GAP domain, GRAF1 can also affect certain small G proteins such as RhoA and Cdc42 (Taylor et al., 1999; Shibata et al., 2001; Jelen et al., 2009).

Due to its multi-domain architecture, GRAF1 has numerous functions that include (1) localizing onto membranes (2) interacting with membrane-shaping proteins, such as Dynamin, and (3) activating RhoA/Cdc42 small G-proteins, thereby indirectly impacting cytoskeleton-related processes. In addition, GRAF1 interacts with Focal Adhesion Kinase (FAK) to regulate cell spreading by remodeling microdomains and the cytoskeleton at podosomes, leading to membrane turnover during morphological adjustments (summarized in Model Figure 8A) (Hildebrand et al., 1996; Doherty et al., 2011).

We propose a novel function for GRAF1 and hypothesize that in addition to its previously described regulatory roles, GRAF1 might be involved in TRE vesiculation. Our study indicates that GRAF1 interacts with both MICAL-L1 and EHD1 to regulate TRE vesiculation and mediate endocytic recycling.

## Methods and materials

### Antibodies

The affinity-purified rabbit polyclonal peptide antibodies directed against the C-terminus of human EHD1 (DLPPHLVPPSKRRHE) were previously described (Caplan et al., 2002). Mouse monoclonal MEM-43 antibody against CD59 was a generous gift of Dr. V. Horejsi (Praha, Czech Republic) (Cai et al., 2011, 2012). The following commercial antibodies were used in this study: mouse anti-GRAF1 (Abcam), mouse anti-HA (Covance), rabbit anti-HA (Signalway), mouse anti-Actin and mouse anti-MICAL-L1 (Novus Biologicals, Inc.), mouse anti-Rab5 (Transduction Laboratories), rabbit anti-Rab11 (US Biologicals), mouse anti-β1 integrin (AbD Serotec), goat anti-mouse horseradish-peroxidase (HRP) (Jackson ImmunoResearch Laboratories, Inc.), donkey anti-rabbit HRP (GE Healthcare), Alexa 568 goat anti-mouse, Alexa 488 goat anti-rabbit and Alexa 568 goat anti-rabbit (Invitrogen). The transfection reagents X-treme GENE 9, GeneExpresso and Oligofectamine were obtained from Roche Applied Science Innovita and Invitrogen, respectively.

### cDNA

GRAF1b and GRAF1c were amplified from HeLa cDNA and human brain cDNA, respectively, using the primers CCGGAATTCGGATGGGGCTCCCAGCGCTCGAGTTCAG and AAGGAAAAAAGCGGCCGCTTAGAGGAACTCCACGTAATTCTCAGG. Amplified GRAF1b and GRAF1c were then cloned into the pHA-CMV vector. HA-GRAF1c (BAR) (1-250aa), HA-GRAF1c (BAR-PH) (1-383aa), HA-GRAF1c (BAR-PH-GAP) (1-663aa) and HA-GRAF1c (R412D) mutants were engineered using PCR directed mutagenesis (Stratagene). GST-GRAF1c was generated as follows: GRAF1c was amplified from HA-GRAF1c by using primers CGCGGATCCATGGGGCTCCCAGCGCTCGAGTTCAG and AAGGAAAAAAGCGGCCGCTTAGAGGAACTCCACGTAATTCTCAGG and cloned into pGEX-6P-2 vector. GST-GRAF1c (BAR-PH) was created by introducing a stop codon following aa 383 in the wild-type GST-GRAF1c.

### Protein purification

GST-GRAF1c and GST-GRAF1c (BAR-PH) were transformed into BL21 bacterial, which was cultured in Super Broth media at 37°C. When the OD reached 0.5, cells were induced with 0.2 mM IPTG and cultured at 18°C overnight. Cells were then lysed by French Press. GST-GRAF1c and GST-GRAF1c (BAR-PH) were purified from the lysates using Glutathione S-transferase beads. EHD1 was cloned into a pCOLD-GST vector and purified as described in Hayashi and Kojima (2008).

### Gene knockdown by silencing RNA (siRNA) and rescue experiments

Four specific oligonucleotides directed at GRAF1 (GCACTACTGTACATATCAA, CAAGGGCTGTATCGAATTG, GAACGGATACGGATGATTG, GGAGAAACGTCACTTTGGA), using On-Target SMART pool (Dhamacon), were transfected into HeLa cells with Oligofectamine (Invitrogen) for 72 h to knockdown endogenous human GRAF1. Mock-SiRNA was essentially the same treatment as for SiRNA-treated cells, excluding the oligonucleotide(s). The siRNA-resistant HA-GRAF1 was engineered using QuickChange site directed mutagenesis. The specificity of the knockdown was confirmed by rescue experiments: the siRNA-resistant HA-GRAF1 was transfected into GRAF1-siRNA-treated cells by using X-treme GENE 9, 48 h after the start of siRNA treatment.

### Immunoblotting

HeLa cells were harvested and lysed on ice for 30 min in buffer containing 50 mM Tris pH7.4, 150 mM NaCl, 0.5% Triton X-100 and protease inhibitor cocktail (Roche Molecular Biochemicals). The lysate supernatants were obtained after centrifugation. Total protein level in the lysate was quantified by Bio-Rad protein assay (Bio-Rad laboratories, Inc.) for equal protein loading on gels in siRNA experiments. Samples were resolved by 8% SDS-PAGE.

### Co-immunoprecipitation

HeLa cells growing on 100 mm dishes were transfected with HA-tagged proteins by using GeneExpresso. After 20 h, cells were lysed for 1 h in buffer containing 50 mM Tris pH7.4, 150 mM NaCl, 0.5% Triton X-100 and protease inhibitor cocktail. Cell lysates were incubated with either mouse anti-MICAL-L1 antibody or rabbit anti-EHD1 antibody at 4°C overnight, followed by incubation with protein L agarose beads (Fisher Thermo Scientific) and Rabbit IP Matrix beads (Santa Cruz Biotechnology) for 3 h at 4°C, respectively. Beads were washed in buffer containing 50 mM Tris pH7.4, 150 mM NaCl, 0.1% Triton X-100 4 times, and proteins were eluted using 4×SDS loading buffer. Samples were subjected to 8% SDS-PAGE, followed by blotting with appropriate antibodies.

### Immunofluorescence, CD59 uptake assay and β1 integrin pulse-chase assay

HeLa cells were grown on cover slips, occasionally transfected with X-treme GENE 9 or oligofectamine, as noted, and fixed with 4% (v/v) paraformaldehyde in PBS as described previously (Cai et al., 2012). Fixed cells were incubated with primary antibodies prepared in staining solution [0.2% saponin (w/v) and 0.5% (w/v) BSA in PBS] for 1 h at room temperature. After washes with PBS, cells were incubated with the appropriate fluorochrome-conjugated secondary antibody mixture in staining solution for 30 min at room temperature. All images were acquired using a Zeiss LSM 5 Pascal confocal microscope (Carl Zeiss) with a 63x 1.4 numerical aperture objective with appropriate filters. For CD59 uptake assay, cells in complete medium were pulsed with anti-CD59 antibody for 15 min at 37°C, followed by 1 min incubation with acid stripping buffer (0.5 M NaCl and 0.5% acetic acid, pH3.0) to remove surface-bound CD59 antibody. Cells were fixed after stripping and stained with appropriate secondary antibodies. For β1 integrin pulse-chase assay, cells were pulsed with anti-β1 integrin for 1 h in complete media at 37°C, followed by 1 min acid stripping to remove surface-bound β1 integrin antibody. Cells were then either fixed directly (pulse only) or “chased” in complete media for 4 h to allow β1 to recycle back to the plasma membrane (pulse and chase). After chase, cells were acid stripped again to remove β1 integrin which had recycled back to the plasma membrane.

### Semi-permeabilization assay

This assay was developed (Cai et al., 2013) and modified from the methods described by Balch (Beckers et al., 1987) and Aridor (Long et al., 2010). Briefly, HeLa cells were grown on coverslips. When the cell confluence reached 80–90%, cytosol was extracted with 20 μg/ml digitonin for 40 s at room temperature. After three times washing in KHM buffer containing 110 mM KOAc, 20 mM HEPES, and 2 mM MgOAc, pH 7.2, semi-permeabilized cells were incubated with GST proteins in the presence of 25 mM HEPES, 115 mM KCl, 2.5 mM MgCl_2_, 1 mM ATP, 5 mM creatine phosphate and 0.2 IU of creatine phosphokinase for 30 min at 37°C. Cells were then washed with KHM buffer for three times, followed by fixation. Fixed cells were stained with anti-MICAL-L1 antibody.

### Quantification of MICAL-L1 tubules by IMAGEJ

In the IMAGEJ software, background from the images was removed by adjusting the threshold. The size of particles measured was set between 2 and 150 μm^2^. Every MICAL-L1-containing particle in this range was counted. Ten fields (images) from each treatment were analyzed.

### Statistical analysis

One-Way ANOVA was used to calculate statistical significance, and all graphs show standard error bars.

### Selective yeast two-hybrid assays

Yeast two-hybrid studies were performed as previously described (Giridharan et al., 2013). The *Saccharomyces cerevisiae* strain AH109 was co-transformed with mentioned constructs using lithium acetate procedure and allowed to grow at 30 C after streaking on plates lacking leucine and tryptophan. Once the colonies grow large enough, an average of three to four colonies were chosen, suspended in water, equilibrated to same optical density at 600 nm and replated on plates lacking leucine and tryptophan (+His) as well as plates also lacking histidine (−His).

## Results

The multi-domain-containing protein, GRAF1, is involved in a variety of processes including membrane-shaping, small G-protein signaling, clathrin-independent endocytosis and cell spreading (Doherty and Lundmark, 2009). Because it contains both a BAR and PH domain, and has a role in membrane bending similar to that of other BAR-containing proteins, and because it localizes to tubular endosomes (Lundmark et al., 2008), we posited that GRAF1 may be involved in the biogenesis of TREs.

Recently, a studies from our lab has demonstrated the role of two key proteins in TRE biogenesis: Syndapin2 and MICAL-L1 (Giridharan et al., 2013), along with the C-Terminal EHD protein, EHD3 (Cai et al., 2013). As does GRAF1, Syndapin2 has a membrane-bending BAR domain, whereas MICAL-L1 has 14 PRDs, short motifs known to interact with proteins containing SH3 domains. Since GRAF1 possesses a SH3 domain (see Figure 1A), we hypothesized that GRAF1 and MICAL-L1 might function together on MICAL-L1-enriched TRE. GRAF1 has 3 alternative splicing products, GRAF1a, b, and c, comprised of 851, 796, and 759 amino acids, respectively (Figure 1A). We first assessed which alternatively spliced GRAF1 product is expressed endogenously in HeLa cells. As shown in Figure 1B, GRAF1b RNA transcripts were abundant in a HeLa cDNA library, whereas we were unable to detect GRAF1a transcripts (Figure 1B). However, since it is not known in which cells and/or tissues GRAF1a is expressed (leaving us without a “positive control” for our detection system), we cannot rule out the possibility that GRAF1a RNA might be expressed in HeLa cells. On the other hand, GRAF1c was the only protein detected (Figure 1C, left lane). This was verified by comparing overexpressed HA-GRAF1b and c, with endogenously expressed GRAF1 in non-transfected HeLa cells (Figure 1C; middle panel). Endogenous GRAF1c protein expression was almost completely knocked-down by siRNA, using specific oligonucleotides (Figure 1C; right panel). Actin was used as a control for equal protein loading.

To assess the potential interaction between MICAL-L1 and GRAF1, HeLa cells were transiently transfected with either HA-GRAF1c, or with a negative control, HA-MAPK (Mitogen-Activated Protein Kinase). The latter control was chosen because it was an unrelated protein with an identical HA-tag to HA-GRAF1c, and its expression was similar to that of HA-GRAF1c. As shown in Figure 1D (left panel), endogenous MICAL-L1 pulled-down HA-GRAF1c but not HA-MAPK (Figure 1D; right panel). Additional controls were done immunoprecipitating with Protein L beads but without the anti-MICAL-L1, further confirming the specificity of this interaction (Figure S1D). However, data from selective yeast two-hybrid assays lead us to suggest that this interaction is likely indirect (Figure S1A).

### MICAL-L1, GRAF1, and EHD1 are components of a complex

**Figure 2:**
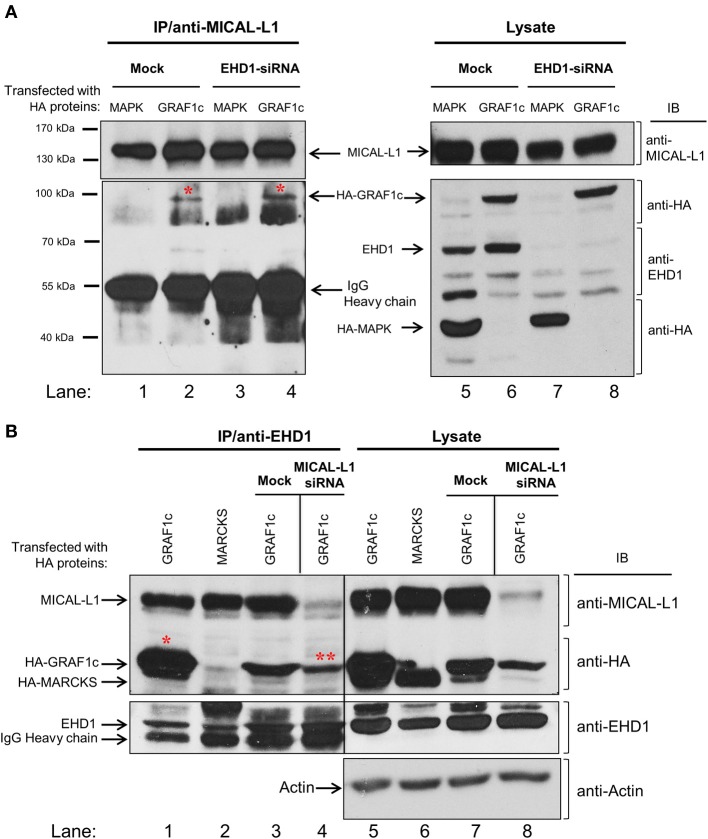
**GRAF1c, MICAL-L1, and EHD1 reside in a common complex. (A)** HeLa cells were either mock-treated or treated with EHD1-siRNA for 72 h. In the final 48 h of the siRNA treatment, cells were transfected with either HA-MAPK (negative control) or HA-GRAF1c. Cells were then lysed with 0.5% Triton X-100 (right panel shows input). Lysates (left panel) were incubated with anti-MICAL-L1 antibody. Protein L beads were added to pull-down MICAL-L1 antibody and elution was done by adding SDS loading buffer. Samples were subjected to 8% SDS-PAGE, followed by blotting with anti-EHD1, anti-HA, and anti-MICAL-L1 antibodies. **(B)** Cells were either transfected with HA-MARCKS (negative control), or HA-GRAF1c, and then either mock-treated or treated with MICAL-L1-siRNA (right panel shows input). Rabbit IP Matrix beads were added to the mixture of cell lysate and EHD1 antibody for 3 h at 4°C. Proteins were eluted and subjected to SDS-PAGE, followed by blotting with anti-HA, anti-MICAL-L1, anti-EHD1, anti-MICAL-L1, and anti-Actin antibodies. Red stars indicate GRAF1c pulled down by either MICAL-L1 or EHD1.

The apparent indirect interaction between GRAF1 and MICAL-L1 led us to query whether these proteins might be linked by a common interaction partner. We have previously demonstrated that MICAL-L1 interacts with EHD1 (Sharma et al., 2009), and we now asked whether EHD1 was necessary for the MICAL-L1/GRAF1 interaction. To this aim, HeLa cells were either mock-treated or depleted of EHD1 by siRNA. The cells were then transfected with either HA-GRAF1 or HA-MAPK (negative control). Lysates from these cells (Figure 2A; right panel) demonstrate that HA-MAPK, HA-GRAF1, endogenous MICAL-L1 and EHD1 were readily detected by their respective antibodies. The lysates also confirmed that EHD1 expression was completely inhibited by EHD1-siRNA. Under these conditions, anti-MICAL-L1 antibody was used for immunoprecipitation, and the presence of HA-GRAF1 or HA-MAPK was tested in the immunoprecipitate. As demonstrated (Figure 2A; left panel), GRAF1 (but not the MAPK negative control) was co-immunoprecipitated by anti-MICAL-L1 antibody in *both* mock-treated cells and cells EHD1-depleted cells (red asterisks). Our data promote the notion that EHD1 is *not* required for the interaction between MICAL-L1 and GRAF1.

The possibility that the interaction between MICAL-L1 and GRAF1 represents a multi-protein complex that includes EHD1 was then tested. MICAL-L1-depleted cells were transfected with cDNA coding for HA-MARCKS (Myristoylated alanine-rich C-kinase substrate; negative control) or HA-GRAF1 (Figure 2B). HA-MARCKS was selected as a negative control because it was unrelated to the TRE biogenesis and vesiculation process, because of its HA-tag, and because it has a similar molecular weight to GRAF1c. The efficiency of MICAL-L1 knock-down is demonstrated in lanes 7–8. EHD1, HA-MARCKS, HA-GRAF1, MICAL-L1, and actin (loading control) were detected in the lysate (input) with the corresponding antibodies (Figure 2B; right panel, lanes 5–8). As previously shown, anti-EHD1 readily pulled down endogenous MICAL-L1 (Figure 2B, lanes 1–3) but not HA-MARCKS (Figure 2B, lane 2). In an additional control immunoprecipitation without the specific EHD1 antibody, no HA-GRAF1c was observed (Figure S1E). However, EHD1 precipitated HA-GRAF1 even in the absence of MICAL-L1 (Figure 2B, lane 4; see double asterisks). This interaction also appeared to be indirect, as suggested by data from yeast two-hybrid assays (Figure S1A; right panel) nor is it regulated by the TRE regulator, Syndapin2 (Figure S1B; right panel). Taken together, our data support the localization of GRAF1 to a complex containing both MICAL-L1 and EHD1.

### GRAF1 supports *in vivo* vesiculation of tubular recycling endosomes

**Figure 3:**
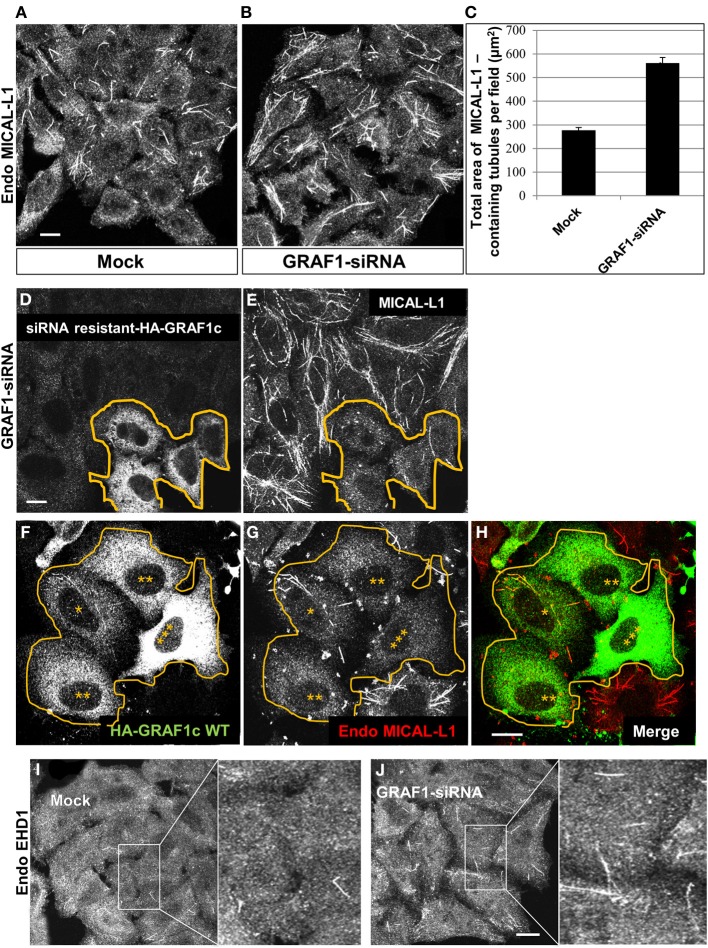
**GRAF1 vesiculates MICAL-L1- and EHD1-decorated membrane tubules. (A)** HeLa cells grown on coverslips were either mock-treated or **(B)** treated with GRAF1-siRNA for 72 h. After fixation, cells were stained with mouse anti-MICAL-L1 antibody for 1 h, followed by Alex 568-goat anti-mouse secondary antibody. **(C)** The total area of MICAL-L1-containing tubules per field in both mock-treated and GRAF1-siRNA-treated cells was quantified by using Image J from three independent experiments **(**represented in **A,B)**. Error bars represent standard error; *p* < 0.001. **(D,E)** siRNA resistant-HA-GRAF1c was re-introduced into GRAF1-depleted cells for 24 h followed by fixation. Fixed cells were then stained with rabbit anti-HA **(D)** and mouse anti-MICAL-L1 antibodies **(E)**, followed by Alexa 488-goat anti-rabbit and Alexa 568-goat anti-mouse secondary antibodies. **(F)** Cells transfected with HA-GRAF1c are shown at variable expression levels (3 asterisks designate the highest expression level). **(G)** These transfected cells (orange line) are stained with anti-MICAL-L1. Note that TRE tend to disappear in the HA-GRAF1c high expressing cells. **(H)** Merged image displaying staining for HA-GRAF1c (green) and endogenous MICAL-L1 (red). **(I,J)** Endogenous EHD1 was detected in either mock-treated **(I)** or GRAF1-depleted cells **(J)**. Bar, 10μm.

Endogenous MICAL-L1 decorates TRE and plays an essential role in their biogenesis by recruiting “tubulators” and “vesiculators” (Giridharan et al., 2013). Support for the involvement of GRAF1 in the biogenesis of TRE was obtained using MICAL-L1 as a marker for these structures. As shown in Figure 3A, MICAL-L1-decorated TRE are readily detected in untreated HeLa cells. However, upon GRAF1-depletion, there is a striking increase in the length and number of TRE (Figure 3B). Quantification of the total area of MICAL-L1-containing TRE indicates a two fold increase per image field upon GRAF1-depletion (Figure 3C). While the total level of MICAL-L1, as tested by immunoblot, is not altered by the GRAF1-knockdown (Figure S1C), the increased number and overall area of TRE at GRAF1-knockdown suggest a potential role for GRAF1 in vesiculating these endosomes.

To ascertain that these results can be directly attributed to GRAF1 knock-down (as opposed to off-target effects), we transfected a siRNA-resistant HA-GRAF1 into the depleted cells (in Figure 3D, boundary marked with an orange line) and analyzed the levels of TRE. Expression of the resistant HA-GRAF1 construct was confirmed (see Figure S2D). As demonstrated, transfected cells in Figures 3D,E had fewer and shorter TRE compared to the neighboring GRAF1-depleted cells (Figure 3E), confirming that indeed GRAF1 is responsible for the phenotype observed.

Whereas GRAF1-knockdown led to inefficient TRE vesiculation, over-expression of HA-GRAF1 induced the opposite effect, causing extensive vesiculation (Figures 3F–H). In Figure 3F a group of transfected cells expressing HA-GRAF1 at variable levels is observed (denoted by asterisks, with 3 asterisks representing the highest level of GRAF1 expression). In Figure 3G, the degree of TRE vesiculation is observed in these transfected cells; in low-expressing HA-GRAF1 cells, a small number of TRE were still visible, but at medium-to-high expression levels, TRE were absent due to extensive vesiculation.

TRE are enriched in PA, which selectively recruits MICAL-L1 and Syndapin2 (Giridharan et al., 2013). To determine whether GRAF1-decorated membrane tubules also contain PA, we co-transfected HA-GRAF1 (at low expression) along with GFP-Spo20p, a well-characterized PA-binding protein (Zeniou-Meyer et al., 2007), (Figures S2A–C; see insets). Their partial co-localization on tubular structures further supports the notion of GRAF1 association with TRE *in vivo*.

Endogenous EHD1 localizes to a wide range of vesicular and tubular membranes within the cell, including TRE (Caplan et al., 2002; Cai et al., 2011), albeit to a lesser degree than MICAL-L1, presumably because it begins to induce vesiculation of these membranes. However, as demonstrated, GRAF1-depletion induced an increase in endogenous EHD-decorated TRE (Figures 3I,J; see insets), further supporting the involvement of GRAF1 in the process of vesiculation.

### GRAF1 promotes vesiculation of incoming and outgoing endosomes

Since GRAF1-depletion affects EHD1-decorated endosomes, this prompted us to further investigate the type of endosomes that serve as target membranes for GRAF1 action. To this aim, in-bound vesicles were visualized by following the uptake of the endogenous GPI-anchored protein (GPI-AP), CD59. Previous studies have demonstrated that GFP-GPI, a transfected GPI-AP model protein, traverses GRAF1-containing CLICs (Lundmark et al., 2008). Endogenous CD59 is a typical cargo of the clathrin-independent pathway that associates with micro-domains enriched with cholesterol and sphingolipids (Stulnig et al., 1998). In mock- and GRAF1-siRNA-treated HeLa cells, anti-CD59 antibody was incubated with cells to allow binding to surface-CD59 and internalization of antibody-receptor complexes. Remaining anti-CD59 on the cell surface was stripped with a short acid-wash, to allow examination exclusively of internalized CD59.

**Figure 4:**
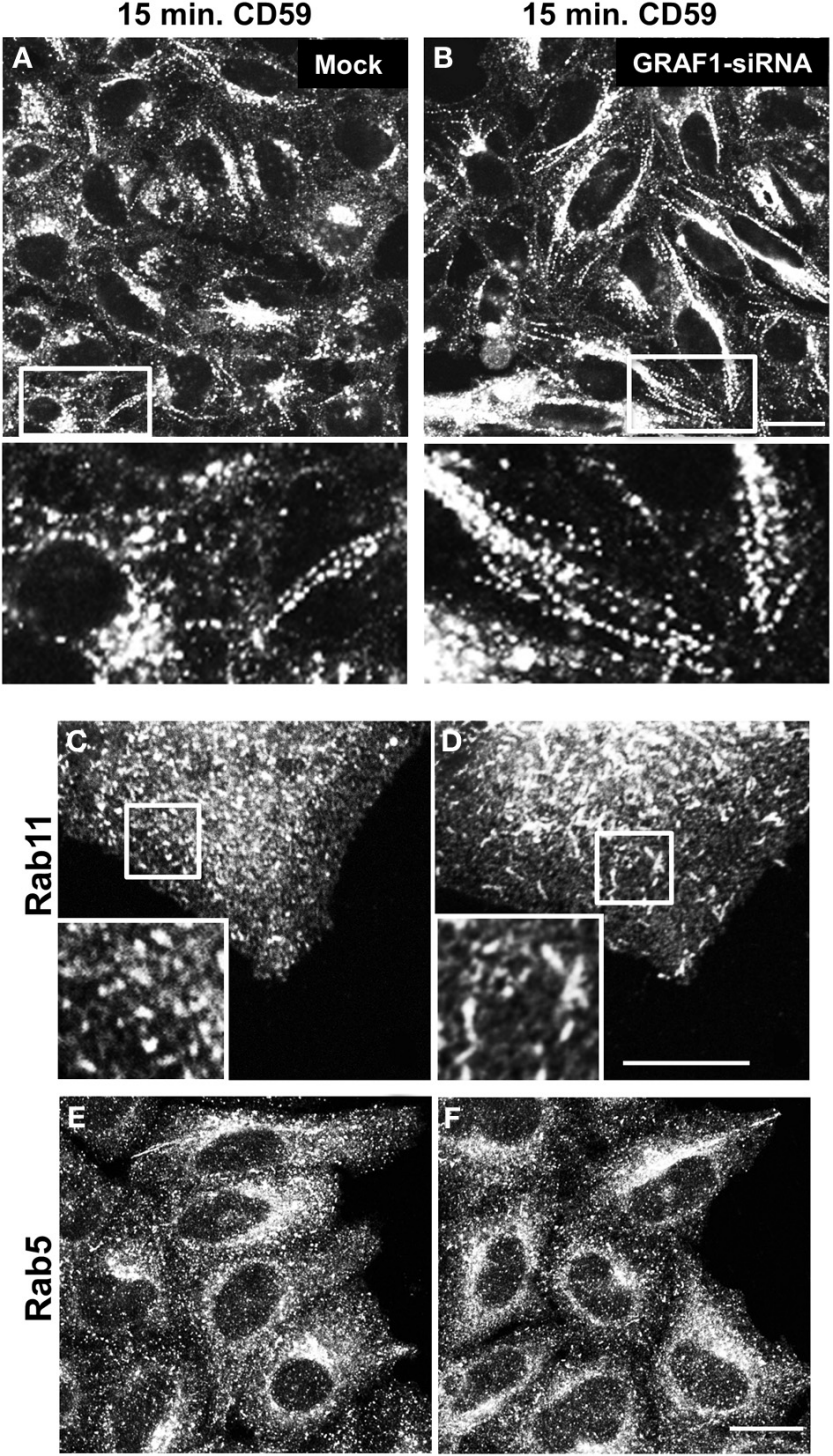
**GRAF1-depletion induces hyper-tubulation of CD59-containing endosomes and Rab11-positive recycling endosomes**. Cells grown on coverslips were either mock-treated **(A,C,E)** or treated with GRAF1-siRNA **(B,D,F)** for 72 h. **(A,B** and insets**)** Cells were incubated with mouse anti-CD59 antibody for 15 min. At 37°C, followed by a 1 min. acid stripping and fixation. Fixed cells were stained with Alex 568-goat anti mouse secondary antibody. After siRNA-treatment, cells were fixed and stained with either anti-Rab11 antibody **(C,D)** or anti-Rab5 antibody **(E,F)**, followed by appropriate secondary antibodies. Bar, 10μm.

We have previously shown that following 15 min. of incubation with anti-CD59 antibody, CD59 is primarily localized to tubular endosomes that are on route to the endocytic recycling compartment (ERC) (Cai et al., 2011, 2012), and therefore used this incubation time to study incoming tubules. Compared to mock-treated cells (Figure 4A), incoming endosomes containing CD59 were extensively tubular upon GRAF1-depletion (Figure 4B; see insets with higher magnification). Note that the bead-like pattern of CD59 is typically obtained post-fixation (Cai et al., 2011).

We next addressed the potential involvement of GRAF1 in the shaping of two well-established endocytic organelles: sorting endosomes (SE) and recycling endosomes (RE). To this aim, we addressed the distribution of the small GTP-binding proteins, Rab5 and Rab11 (respectively) in mock- vs. GRAF1-depleted cells. In Figures 4C,D, Rab11-associated RE were mostly punctate in mock-treated cells, but displayed a degree of elongation (short tubules) upon the loss of GRAF1. On the other hand, Rab5-decorated SE did not undergo discernible changes (Figures 4E,F). Taken together, GRAF1 affects membrane-vesiculation of clathrin-independent incoming endosomes as well as RE, but not Rab5-sorting endosomes.

### Exit of β1 integrin from the endocytic recycling compartment is delayed in the absence of GRAF1

**Figure 5:**
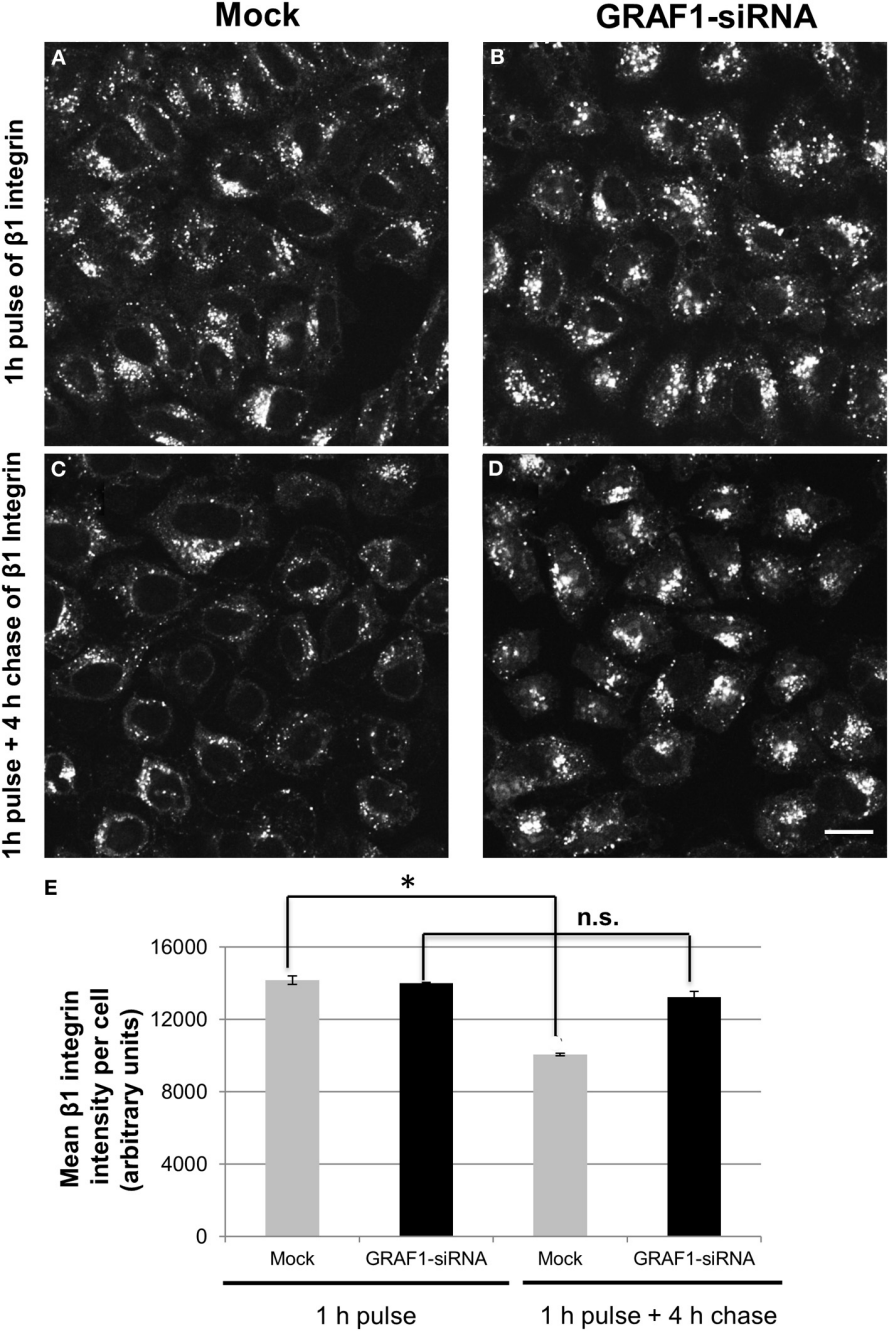
**The recycling of β1 integrin is delayed upon GRAF1-depletion. (A)** Cells grown on coverslips were either mock-treated or **(B)** treated with GRAF1-siRNA for 72 h. Cells were incubated with mouse anti-β1 integrin for 1 h at 37°C, followed by a short acid strip and either fixed directly **(A,B)** or “chased” in complete media for 4 h, followed by a second acid strip and fixation **(C,D)**. Fixed cells were stained with Alexa 568-goat anti-mouse secondary antibody **(A,D)**. **(E)** The intensity of remaining β1 integrin per cell in both mock-and siRNA treatments was quantified by Image J from three independent experiments **(E)**. ^*^p < 0.001. n.s., not significant. Error bars depict standard error. Bar, 10μm.

Vesiculation gives rise to mobile transport vesicles, which move along microfilaments, tethered to motor proteins, and unload their cargo at their target destination, and is thus crucial for the process of recycling. Therefore, since MICAL-L1- and Rab11-containing RE are target-vesicles for GRAF1, we hypothesized that GRAF1-induced vesiculation might directly affect the exit of cargo molecules from the recycling compartment either to the plasma membrane (PM), or to the intracellular degradative pathways. β1 integrins are transmembrane proteins that mediate the attachment of the cells to the extracellular matrix. They can be internalized independently of clathrin (Altankov and Grinnell, 1995; Ylanne et al., 1995), or via clathrin-coated pits (Nishimura and Kaibuchi, 2007; Ezratty et al., 2009), and recycle through a MICAL-L1-mediated pathway (Sharma et al., 2009). Accordingly, we assessed the involvement of GRAF1 in β1 integrin exit from the recycling compartment. Cells were mock-treated, or treated with GRAF1-siRNA, then incubated with β1 integrin antibody for 1 h to allow internalization, followed by a short acid-strip to remove remaining non-internalized antibody. While similar amounts of β1 integrin were internalized upon GRAF1-depletion (compare Figures 5B to 5A; quantified in 5E, 1 h pulse), a clear delay in β1 integrin exit from the recycling compartment was evident upon GRAF1-depletion (compare Figures 5D to 5C; quantified in 5E, 4 h chase). Indeed, we observed a ~25% delay in the rate of β1 integrin recycling to the PM and/or degradation in GRAF1-depleted cells (Figure 5E).

### The RhoA-gap domain of GRAF1 is required for vesiculation of tubular recycling endosomes

**Figure 6:**
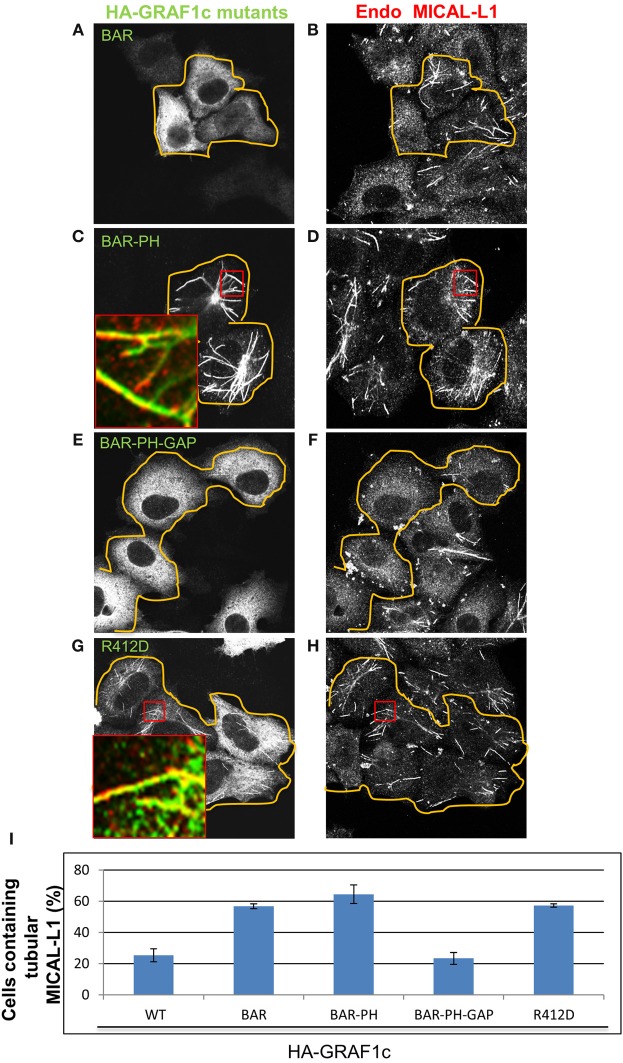
**The GAP domain of GRAF1c is required to promote vesiculation of MICAL-L1-containing tubular endosomes**. Cells grown on coverslips were either transfected with **(A,B)** the HA-GRAF1c BAR domain (orange lines indicate the transfected cells), **(C,D)** the HA-GRAF1c BAR-PH domains, **(E,F)** the HA-GRAF1c BAR-PH-GAP domains, or **(G,H)** the mutant HA-GRAF1c (R412D) for 12 h. Cells were then fixed and stained with rabbit anti-HA and mouse anti-MICAL-L1 antibodies, followed by Alexa-488 goat anti-rabbit and Alex-568 goat anti- mouse antibodies. **(I)** Quantification of the percentage of cells containing MICAL-L1-decorated tubular recycling endosomes, derived from three independent experiments. Error bars denote standard error. Bar, 10μm.

To identify the domain(s) of GRAF1 responsible for vesiculation, we transfected a series of truncation mutants or HA-GRAF1 point mutations and used the morphology of MICAL-L1-containing TRE as a readout to measure vesiculation *in vivo*. A truncated HA-GRAF1 form, containing only the BAR domain (Figure 6A; quantified in Figure 6I), displayed a cytoplasmic distribution and had no effect on MICAL-L1-decorated endosomes (Figure 6B). A form of GRAF1 comprised of only the BAR+PH domains (Figure 6C), extensively localized to MICAL-L1-decorated TRE (Figure 6D, see inset; quantified in Figure 6I). These findings support previous observations by Lundmark et al. (2008) characterizing the BAR-PH region of GRAF1 as an inducer of static tubular invaginations, incapable of undergoing fission. Thus, in analogy with the role of the PH domain in linking GRAF1 to PIP2 at the plasma membrane (Lundmark et al., 2008), our results support a role for this domain in recruiting GRAF1 to TRE, which also contain PIP2 (Jovic et al., 2009).

Nevertheless, when the Rho-GAP domain was added to the BAR+PH domains (without the SH3 domain), transfected cells were now devoid of TRE compared to neighboring untransfected cells (BAR+PH+GAP; Figures 6E,F; quantified in Figure 6I). These experiments led to the notion that the Rho-GAP domain may have the ability to vesiculate membranes independently of its SH3 domain, despite the fact that the SH3 domain interacts with Dynamin1 and 2 *in vitro* (Lundmark et al., 2008). Thus, it is possible that the recruitment of Dynamin via the GRAF1 SH3 domain may not be required for TRE vesiculation *in vivo*.

To further address the ability of the Rho-GAP domain to vesiculate TRE *in vivo*, site-directed mutagenesis was used to inactivate the Rho-GAP domain (R412D) of full-length HA-GRAF1, giving rise to a “dead-GAP” mutant, incapable of stimulating GTP hydrolysis by Cdc42 (Doherty et al., 2011). At both high and low expression levels of the R412D GRAF1 mutant (Figure 6H), MICAL-L1-decorated TRE were preserved (Figure 6I; quantified in Figure 6J) and comparable to those in untransfected cells. Moreover, the association of GRAF1 (BAR+PH) with TRE did not require the TRE hub protein, MICAL-L1 or Syndapin2 (Figures S3A–E). Taken together, these data provide evidence for the involvement of the Rho-GAP domain of GRAF1 in TRE vesiculation *in vivo*.

### GRAF1 and EHD1 vesiculate tubular recycling endosomes synergistically in a semi-permeabilized cell system

*In vitro* experiments with liposomes have demonstrated that N-BAR-domain-containing proteins shape membranes into tubules, or into small vesicles, depending on the local concentration of the BAR domain, its degree of oligomerization and the membrane density (Peter et al., 2004; Ayton et al., 2009). Our *in vivo* experiments in this study implicate GRAF1 in the vesiculation process; such membrane sculpting activity might be assisted by other proteins that associate with GRAF1. Since TRE nonetheless underwent scission *in vivo* when an over-expressed GRAF1 protein lacking its SH3 domain was introduced into cells (Figures 6E,F), we speculated that the SH3-interacting scission protein, Dynamin, might be dispensable for TRE vesiculation. Accordingly, we turned to investigate the participation of other putative “co-vesiculators” in this process.

Members of the mammalian EHD family (which do not contain a BAR domain) were likewise implicated in oligomerizing and remodeling membranes *in vivo* and *in vitro* (Daumke et al., 2007; Moren et al., 2012; Giridharan et al., 2013). Human EHD1, which decorates a wide range of endosomes, including TRE, demonstrated vesiculation-capabilities *in vivo*, upon ATP hydrolysis (Cai et al., 2012, 2013). It is of interest that a common EHD1/EHD3 ortholog in the lamprey (I-EHD), which displays 83% identity to both human EHD, cooperates with dynamin to promote vesicle budding and to prevent the generation of long tubular structures (Jakobsson et al., 2011). However, depletion of dynamin2 did not lead to elongation of TRE (Figures S3F,G), as might be expected if dynamin2 played an essential role in TRE vesiculation. Indeed, the loss of dynamin appeared to destabilize the tubules (Figures S3F,G). While the reason for this phenotype remains unknown, it is apparent that dynamin2 is not likely to be a major player in the scission of TRE alongside EHD1 and GRAF1.

**Figure 7:**
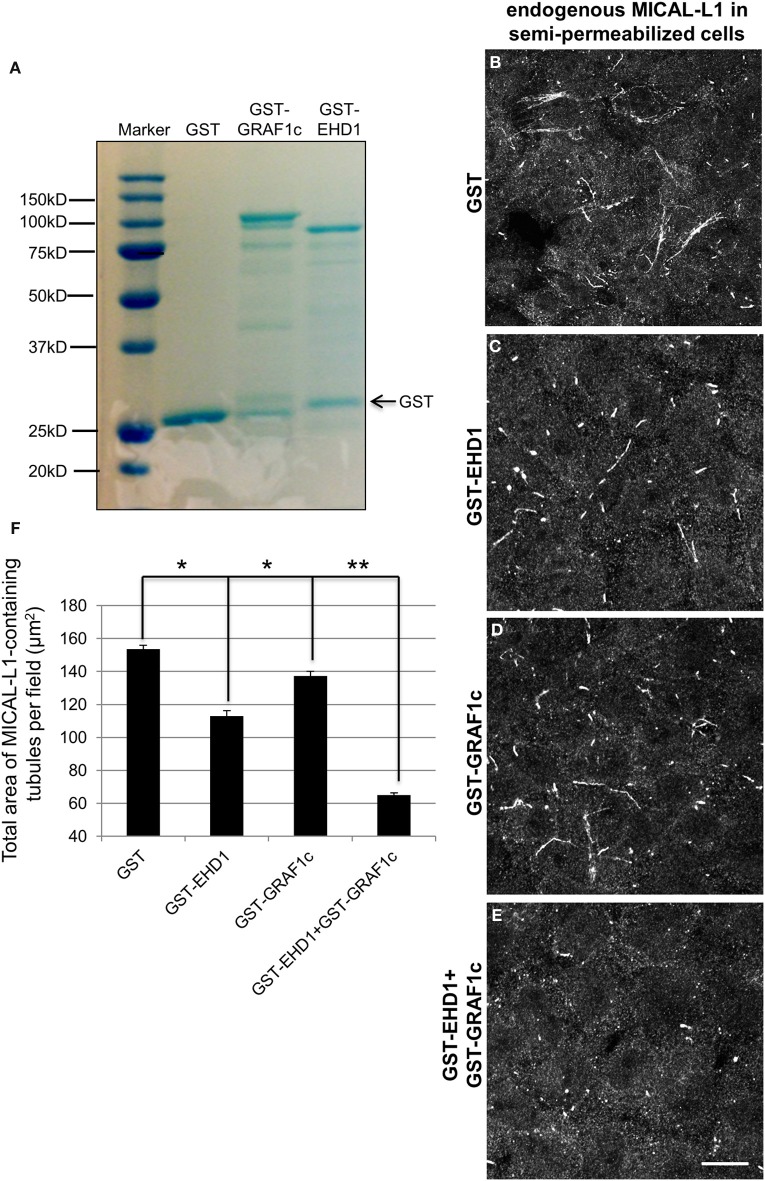
**GRAF1c and EHD1 vesiculate MICAL-L1-containing tubules synergistically in a semi-permeabilized cell system. (A)** Purified GST only, GST-GRAF1c, and GST-EHD1 were detected by SDS-PAGE and Coomassie Blue staining. **(B–E)** Cells were permeabilized with 20 μg/ml digitonin for 40 s at room temperature. Permeabilized cells were then either incubated for 30 min. at 37°C with **(B)** GST-only, **(C)** GST-EHD1, **(D)** GST-GRAF1c, or **(E)** GST-EHD1 and GST-GRAF1c. After fixation, the remaining TRE were stained with anti-MICAL-L1 antibody **(B–E)**. **(F)** The total area of MICAL-L1-containing tubules per field was quantified by Image J from three independent experiments. ^*^p < 0.005, ^**^p < 0.001. Error Bar denotes standard error. Bar, 10μm.

Since MICAL-L1 binds to both EHD1 and GRAF1 directly, and indirectly, respectively, and because EHD1 also associates with GRAF1 (Figure 2B), we aimed to assess the impact of both GRAF1 and EHD1 on TRE-scission in a newly developed semi-permeabilized cell system (Cai et al., 2013). HeLa cells grown on coverslips were gently perforated with digitonin for 40 s at room temperature and much of the cytosol content was washed away. Upon restoring an ATP-regenerating system, the cells were viable for ~1 h, and MICAL-L1-containing TRE remained partially intact. During this period, purified GST-linked proteins (Figure 7A) were introduced to the semi-permeabilized cells for 30 min at 37°C, and subsequently fixed. TRE morphology was then visualized by staining endogenous MICAL-L1 (Figures 7B–E) and the mean area of MICAL-L1-containing tubules per field was quantified (Figure 7F).

The baseline level of TRE was defined by incubating semi-permeabilized cells with GST-only (Figure 7B). Under these conditions, GST-EHD1 incubation (Figure 7C) decreased the tubulation by 27% (mean square area of TRE from 3 independent experiments is plotted in Figure 7F), leaving numerous short, but somewhat elongated endosomes. This is in line with our previous report (Cai et al., 2013) and our *in vivo* observation attributing a role for EHD1 in TRE vesiculation (Cai et al., 2012). By itself, GST-GRAF1 caused a milder decrease in TRE levels (10%) (Figure 7D and quantified in **7F**). However, the addition of both GST-EHD1 and GST GRAF1 *together* brought about a dramatic loss of TRE (57% decrease, Figure 7E and quantified in **7F**), suggesting that GRAF1 and EHD1 act synergistically to induce TRE vesiculation in a semi-permeabilized cell system. Interestingly, the truncated GST-GRAF1 (BAR+PH) did not alter TRE morphology (Figures S4A–C), possibly due to the absence of as-yet-unidentified cytoplasmic components that are lacking in the semi-permeabilized system, but are present in cells. Overall, these data support a role for the GRAF1 Rho-GAP domain in vesiculation.

## Discussion

### Biogenesis of tubular recycling endosomes

The ERC is considered a morphologically distinct series of membranes from early endosomes and comprises a series of vesicular and tubular structures that enable sorting and recycling of internalized receptors and lipids back to the plasma membrane (Gruenberg and Maxfield, 1995). The mechanisms involved in the generation of the ERC and the TREs that comprise this compartment are only partially understood. Recent studies support the role of BAR domain-containing proteins such as BIN1/Amphiphysin (Pant et al., 2009) and Syndapin2 (Braun et al., 2005; Giridharan et al., 2013) in TRE biogenesis, and as well as MICAL-L1 (Giridharan et al., 2013) and the C-terminal EHD protein, EHD3 (Cai et al., 2013). However, once TREs are generated and cargo has been sorted, for recycling to occur there must be efficient vesiculation of these tubular structures.

In previous studies, we and others have identified EHD1 as an ATPase involved in membrane scission and the generation of recycling vesicles (Jakobsson et al., 2011; Cai et al., 2012). While the addition of purified EHD1 and an ATP regenerating system may be sufficient in a semi-permeabilized cell vesiculation assay, *in vivo* other proteins likely contribute to this process. For example, recruitment of PLA2α to tubular membranes may further contribute TRE vesiculation through the generation of lysophosphatidic acid (Cai et al., 2012). We now provide evidence that GRAF1 contributes to TRE vesiculation.

### Vesiculation of membranes by GRAF1, EHD1 and dynamin2

**Figure 8:**
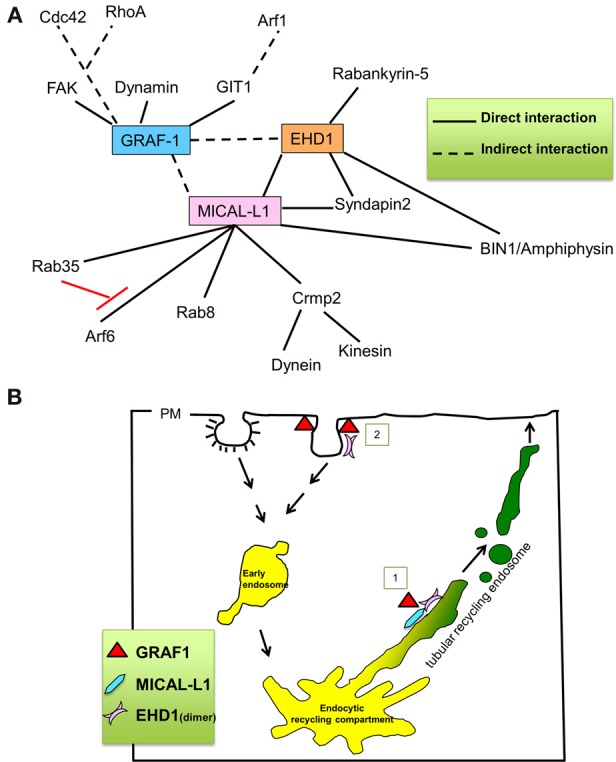
**Proposed role for GRAF1 in mediating vesiculation of tubular recycling endosomes. (A)** Key interaction partners of GRAF1, MICAL-L1 and EHD1. **(B)** Proposed roles for GRAF1 and EHD1 at the plasma membrane, in the clathrin-independent endocytic pathway [pathway 2] [(Cai et al., 2011) and reviewed in Doherty and Lundmark (2009)], and for GRAF1, MICAL-L1 and EHD1 at tubular recycling endosomes [pathway 1]; MICAL-L1 is recruited onto TRE by directly binding to phosphatidic acid. It then recruits EHD1 by a direct interaction and interacts indirectly with GRAF1. EHD1, and GRAF1 then synergistically vesiculate TRE, giving rise to small and motile recycling endosomes.

GRAF1 has been implicated in the control of the poorly characterized CLIC/GEEC endocytic pathway through its ability to regulate the activity of Cdc42 and RhoA (Lundmark et al., 2008) (summarized in Figure 8A). Via its BAR and PH domains, GRAF1 localizes to incoming tubular and vesicular membrane structures, which carry internalized cargo, including bacterial toxins and fluid phase cargo, as well as the model GPI anchored protein, GFP-GPI (Lundmark et al., 2008). Formation of CLIC vesicles requires membrane deformation followed by fission, yet little is known about the driving force for this process. Although dynamin, a fission protein in clathrin-dependent endocytosis, interacts with the SH3 domain of GRAF1 through its PRD domain (Figure 8A), it appears to be dispensable for membrane fission in the CLIC pathway (Doherty and Lundmark, 2009). Consistent with these findings, we also observed that dynamin2 is neither involved in the fission of endosomes containing the GPI-AP, CD59, nor TRE decorated with MICAL-L1. Instead, we have demonstrated that EHD1 mediates this vesiculation process (Cai et al., 2012).

In this study, we show that the GRAF1-depletion and over-expression of GRAF1 induce hypertubulation and vesiculation of recycling tubules, respectively, in a manner analogous to that of EHD1 (Cai et al., 2012). Our data are therefore consistent with the notion that GRAF1 also plays a role in the vesiculation of membrane tubules. Membrane curvature induced by BAR-domain may lead to membrane fission (Peter et al., 2004; Gallop and McMahon, 2005). However, compared with other BAR-containing proteins, the BAR domain of GRAF1 is relatively inefficient in this respect (Doherty and Lundmark, 2009). In agreement with this, the over-expression of the GRAF1-BAR domain did not enhance the vesiculation of recycling tubules (Figures 6A,B). Nor did it enhance vesiculation when expressed together with the PH domain (BAR-PH) (Figures 6C,D). In contrast, when the GAP domain of GRAF1 was transfected into cells, TRE were extensively vesiculated (Figures 6E,F), whereas the GAP-dead mutant was incapable of promoting vesicualtion (Figures 6G,H). Overall, our study supports the idea that the GAP domain of GRAF1 plays a role in the vesiculation process.

Via its PH domain, GRAF1 was previously shown to predominantly bind to PIP2 (Lundmark et al., 2008; Doherty and Lundmark, 2009), which is a major phosphoinositide in the plasma membrane. On the other hand, MICAL-L1 binds selectively to PA, which is enriched on TRE (Giridharan et al., 2013). However, both phosphatidylinositol-4-phosphate and PIP2 are also localized to TRE (Jovic et al., 2009), potentially mediating docking of GRAF1 on tubular and vesicular endosomes. Although GRAF1 seemingly interacts with both MICAL-L1 and EHD1 indirectly (see Figure 8A), MICAL-L1 nevertheless appears to be a major hub that recruits both the components involved in TRE biogenesis (tubulation) as well as vesiculation, potentially allowing synergistic activity between EHD1 and GRAF1 (Figure 7).

Based on our findings, we propose that in addition to GRAF1's characterized roles in CLIC/GEEC regulation and its functions at the plasma membrane (Lundmark et al., 2008; Doherty and Lundmark, 2009; Doherty et al., 2011) (see Model in Figure 8B; pathway number 2, at clathrin-independent invaginations), GRAF1 is also a component of the vesiculation complex comprised of MICAL-L1 and EHD1 on TRE (Figure 8B; pathway number 1, on TRE). Future studies will be needed to determine whether the combined scission activities of EHD1 and GRAF1 are required *in vivo* to overcome energy barriers, or perhaps are needed to effectively regulate the complex process of recycling from the TRE.

### Conflict of interest statement

The authors declare that the research was conducted in the absence of any commercial or financial relationships that could be construed as a potential conflict of interest.

## Supplementary material

Figure S1 **GRAF1c does not interact with MICAL-L1, EHD1, and Syndapin2 by selective yeast two hybrid assays. (A)** The *Saccharomyces cerevisiae* yeast strain AH109 was co-transformed with the indicated GAL4-binding domain (GAL4bd) fusion constructs GAL4bd- GRAF1c and GAL4bd-p53 (control), together with the indicated GAL4 transcription activation (GAL4ad) fusion constructs GAL4ad-EHD1, GAL4ad-MICAL-L1 and GAL4ad-SV40 large T-antigen (control). **(B)** The interaction between GAL4ad-GRAF1c and GAL4bd-Syndapin2 was analyzed. Cotransformants were assayed for their growth on non-selective (+HIS) and selective (-HIS) media. **(C)** GRAF1-depletion does not alter the cellular expression levels of either MICAL-L1 or EHD1. Mock-treated or GRAF1-siRNA-treated cells were subject to immunoblotting with anti-MICAL-L1, GRAF1 (control), EHD1 and actin (loading control). **(D,E)** Cells were transfected with HA-GRAF1c. After 48 h, cells were lysed for 1 h in buffer containing 50 mM Tris pH 7.4, 150 mM NaCl, 0.5% Triton X-100 and protease inhibitors. Cell lysates were incubated with mouse anti-MICAL-L1 antibody or rabbit EHD1 antibody overnight. Protein L beads **(D)** or Rabbit IP Matrix beads **(E)** were added to the mixture of cell lysate and MICAL-L1 or EHD1 antibody for 3 h at 4°C. Beads were washed in buffer containing 50 mM Tris pH 7.4, 150 mM NaCl, 0.1% Triton. Proteins were eluted by adding SDS loading buffer. Samples were subjected to 8% SDS-PAGE, followed by blotting with anti-HA and either anti-MICAL-L1 or EHD1 antibodies.

Figure S2 **GRAF1-containing tubular recycling endosomes are positive for phosphatidic acid. (A–C)** HeLa cells were cotransfected with HA-GRAF1 and the phosphatidic acid-binding yeast protein, GFP-SPO-20. Insets show that SPO-20 localizes in part to GRAF1-containing tubular endosomes. **(D)** Mock- or GRAF1-depleted cells were transfected after 48 h treatment with either wild-type or siRNA-resistant HA-GRAF1c for an additional 24 h. Lysates were then separated by SDS PAGE and immunoblotted with anti-HA or anti-actin antibodies. Bar, 10μm.

Figure S3 **The tubular localization of the GRAF1c BAR-PH domains do not require MICAL-L1 or Syndapin2 expression. (A,B)** Cells grown in 35 mm dishes were either mock-treated, treated with **(A)** MICAL-L1-siRNA or **(B)** Syndapin2-siRNA for 72 h. After lysis, cell lysates were subjected to 8% SDS-PAGE, followed by immunoblotting with anti-MICAL-L1, Syndapin2 and Actin antibodies. **(C–E)** In the last 20 h of siRNA-treatment, cells were transfected with HA-GRAF1c (BAR-PH). After fixation, cells were stained with rabbit anti-HA antibody, followed by Alexa-488 goat anti-rabbit secondary antibody. **(F,G)** Cells were treated with Mock- or Dynamin2-SiRNA for 48 h, and then fixed and stained with anti-MICAL-L1. Efficacy of Dynamin2 depletion was demonstrated by its impact on transferrin uptake (data not shown). Bars, 10μm.

Figure S4 **The GRAF1c BAR-PH domains are unable to vesiculate MICAL-L1-containing tubules. (A)** Purified GST and GST-GRAF1c (BAR-PH) proteins were detected by SDS-PAGE and Coomassie Blue staining. Cells were permeabilized with 20 μg/ml digitonin for 40 s at room temperature. Permeabilized cells were either incubated with **(B)** GST-only and **(C)** GST-GRAF1c (BAR-PH) for 30 min. at 37°C. Cells were then fixed and stained with anti-MICAL-L1 antibody. Bar, 10μm.
