# Supplementary figures and images for: GRAF1 forms a complex with MICAL-L1 and EHD1 to cooperate in tubular recycling endosome vesiculation
Source: Front Cell Dev Biol. 2014 May 27;2:22. doi: 10.3389/fcell.2014.00022 (PMC4214196; doi:10.3389/fcell.2014.00022)

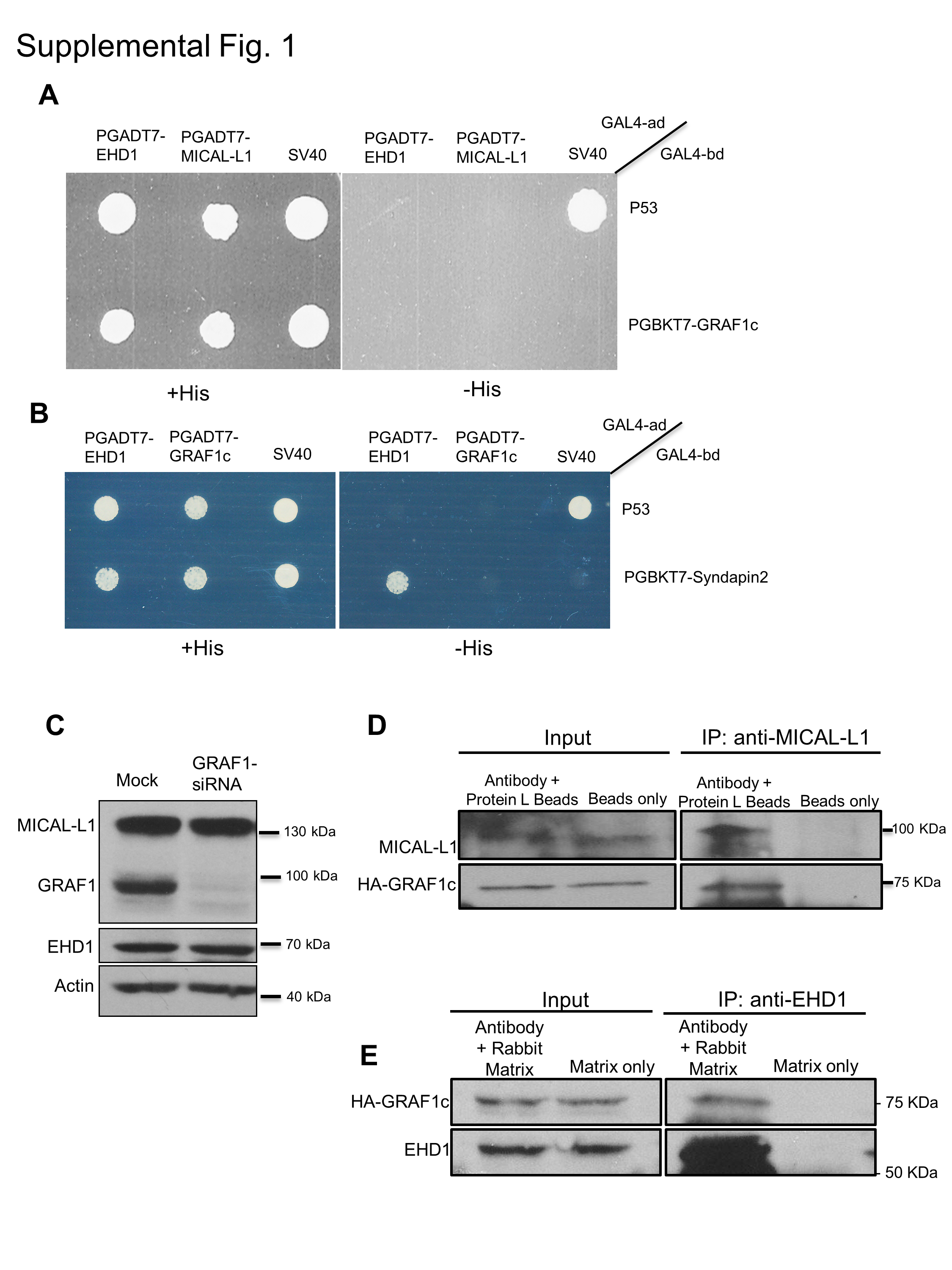

Supplement: Figure S1 — GRAF1c does not interact with MICAL-L1, EHD1, and Syndapin2 by selective yeast two hybrid assays. (A) The Saccharomyces cerevisiae yeast strain AH109 was co-transformed with the indicated GAL4-binding domain (GAL4bd) fusion constructs GAL4bd- GRAF1c and GAL4bd-p53 (control), together with the indicated GAL4 transcription activation (GAL4ad) fusion constructs GAL4ad-EHD1, GAL4ad-MICAL-L1 and GAL4ad-SV40 large T-antigen (control). (B) The interaction between GAL4ad-GRAF1c and GAL4bd-Syndapin2 was analyzed. Cotransformants were assayed for their growth on non-selective (+HIS) and selective (-HIS) media. (C) GRAF1-depletion does not alter the cellular expression levels of either MICAL-L1 or EHD1. Mock-treated or GRAF1-siRNA-treated cells were subject to immunoblotting with anti-MICAL-L1, GRAF1 (control), EHD1 and actin (loading control). (D,E) Cells were transfected with HA-GRAF1c. After 48 h, cells were lysed for 1 h in buffer containing 50 mM Tris pH 7.4, 150 mM NaCl, 0.5% Triton X-100 and protease inhibitors. Cell lysates were incubated with mouse anti-MICAL-L1 antibody or rabbit EHD1 antibody overnight. Protein L beads (D) or Rabbit IP Matrix beads (E) were added to the mixture of cell lysate and MICAL-L1 or EHD1 antibody for 3 h at 4°C. Beads were washed in buffer containing 50 mM Tris pH 7.4, 150 mM NaCl, 0.1% Triton. Proteins were eluted by adding SDS loading buffer. Samples were subjected to 8% SDS-PAGE, followed by blotting with anti-HA and either anti-MICAL-L1 or EHD1 antibodies. [file Presentation1.ZIP › 93240_Caplan_Supplemental_Figure_1.TIF]

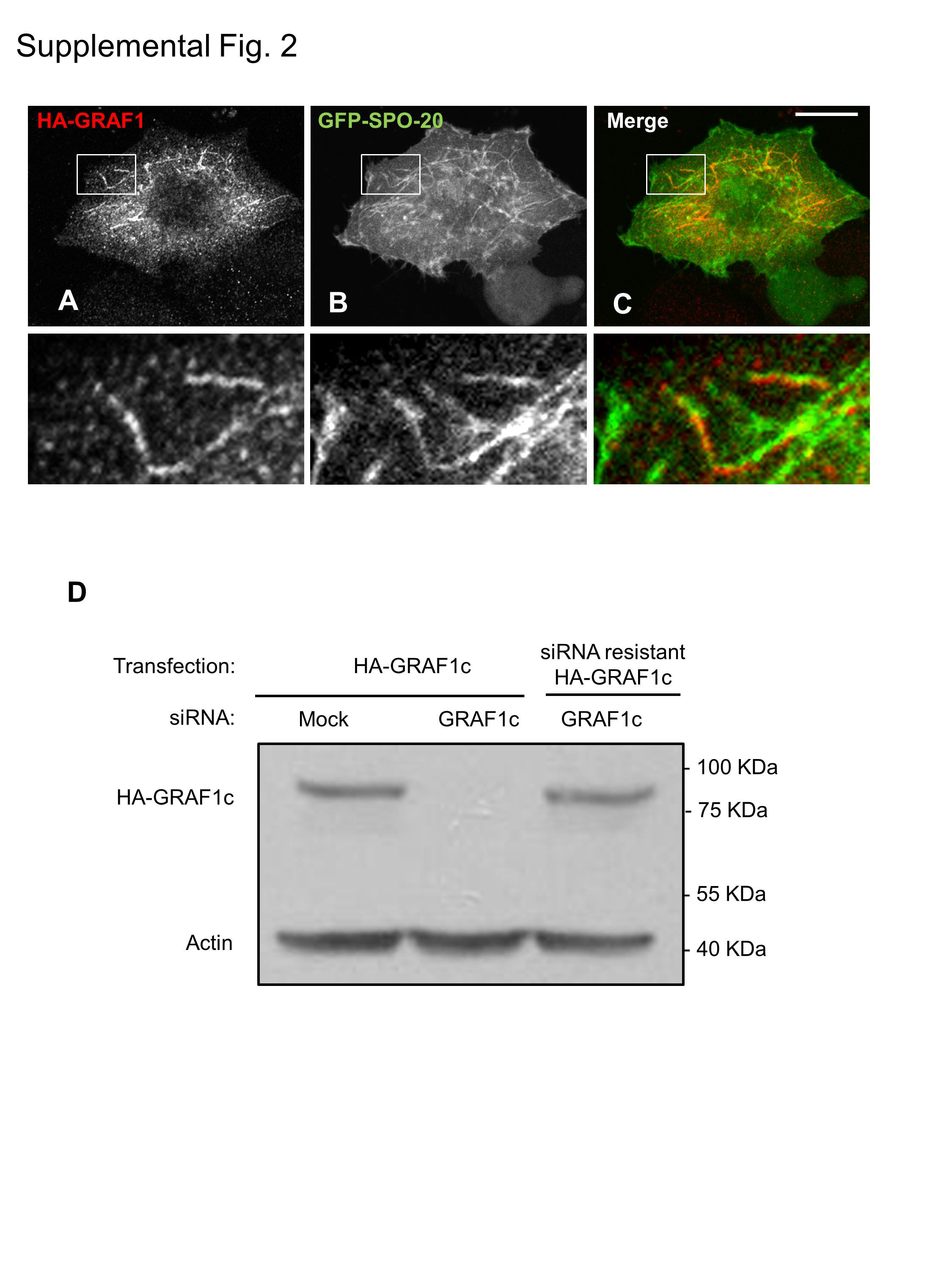

Supplement: Figure S1 — GRAF1c does not interact with MICAL-L1, EHD1, and Syndapin2 by selective yeast two hybrid assays. (A) The Saccharomyces cerevisiae yeast strain AH109 was co-transformed with the indicated GAL4-binding domain (GAL4bd) fusion constructs GAL4bd- GRAF1c and GAL4bd-p53 (control), together with the indicated GAL4 transcription activation (GAL4ad) fusion constructs GAL4ad-EHD1, GAL4ad-MICAL-L1 and GAL4ad-SV40 large T-antigen (control). (B) The interaction between GAL4ad-GRAF1c and GAL4bd-Syndapin2 was analyzed. Cotransformants were assayed for their growth on non-selective (+HIS) and selective (-HIS) media. (C) GRAF1-depletion does not alter the cellular expression levels of either MICAL-L1 or EHD1. Mock-treated or GRAF1-siRNA-treated cells were subject to immunoblotting with anti-MICAL-L1, GRAF1 (control), EHD1 and actin (loading control). (D,E) Cells were transfected with HA-GRAF1c. After 48 h, cells were lysed for 1 h in buffer containing 50 mM Tris pH 7.4, 150 mM NaCl, 0.5% Triton X-100 and protease inhibitors. Cell lysates were incubated with mouse anti-MICAL-L1 antibody or rabbit EHD1 antibody overnight. Protein L beads (D) or Rabbit IP Matrix beads (E) were added to the mixture of cell lysate and MICAL-L1 or EHD1 antibody for 3 h at 4°C. Beads were washed in buffer containing 50 mM Tris pH 7.4, 150 mM NaCl, 0.1% Triton. Proteins were eluted by adding SDS loading buffer. Samples were subjected to 8% SDS-PAGE, followed by blotting with anti-HA and either anti-MICAL-L1 or EHD1 antibodies. [file Presentation1.ZIP › 93240_Caplan_Supplemental_Figure_2.TIF]

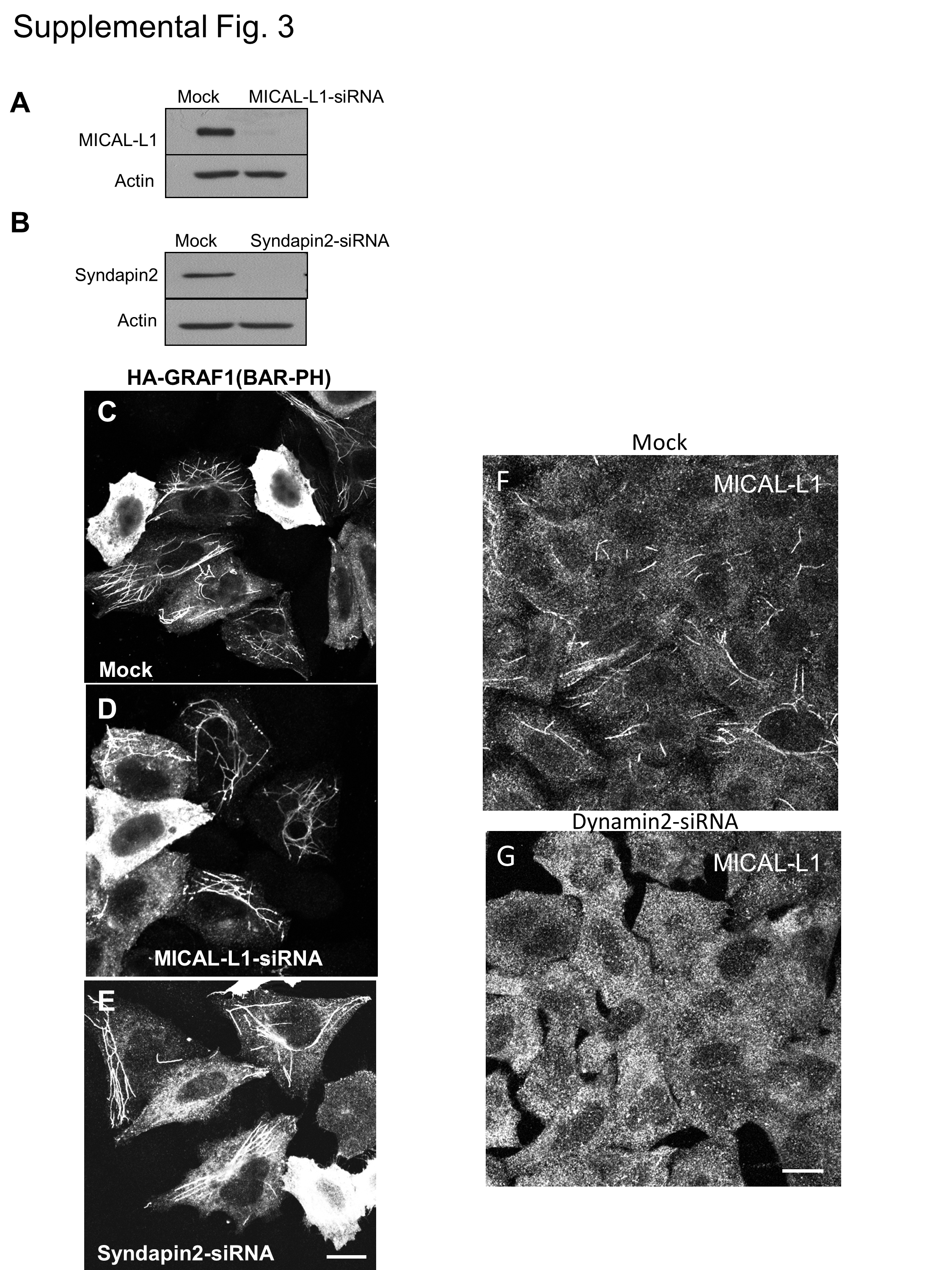

Supplement: Figure S1 — GRAF1c does not interact with MICAL-L1, EHD1, and Syndapin2 by selective yeast two hybrid assays. (A) The Saccharomyces cerevisiae yeast strain AH109 was co-transformed with the indicated GAL4-binding domain (GAL4bd) fusion constructs GAL4bd- GRAF1c and GAL4bd-p53 (control), together with the indicated GAL4 transcription activation (GAL4ad) fusion constructs GAL4ad-EHD1, GAL4ad-MICAL-L1 and GAL4ad-SV40 large T-antigen (control). (B) The interaction between GAL4ad-GRAF1c and GAL4bd-Syndapin2 was analyzed. Cotransformants were assayed for their growth on non-selective (+HIS) and selective (-HIS) media. (C) GRAF1-depletion does not alter the cellular expression levels of either MICAL-L1 or EHD1. Mock-treated or GRAF1-siRNA-treated cells were subject to immunoblotting with anti-MICAL-L1, GRAF1 (control), EHD1 and actin (loading control). (D,E) Cells were transfected with HA-GRAF1c. After 48 h, cells were lysed for 1 h in buffer containing 50 mM Tris pH 7.4, 150 mM NaCl, 0.5% Triton X-100 and protease inhibitors. Cell lysates were incubated with mouse anti-MICAL-L1 antibody or rabbit EHD1 antibody overnight. Protein L beads (D) or Rabbit IP Matrix beads (E) were added to the mixture of cell lysate and MICAL-L1 or EHD1 antibody for 3 h at 4°C. Beads were washed in buffer containing 50 mM Tris pH 7.4, 150 mM NaCl, 0.1% Triton. Proteins were eluted by adding SDS loading buffer. Samples were subjected to 8% SDS-PAGE, followed by blotting with anti-HA and either anti-MICAL-L1 or EHD1 antibodies. [file Presentation1.ZIP › 93240_Caplan_Supplemental_Figure_3.TIF]

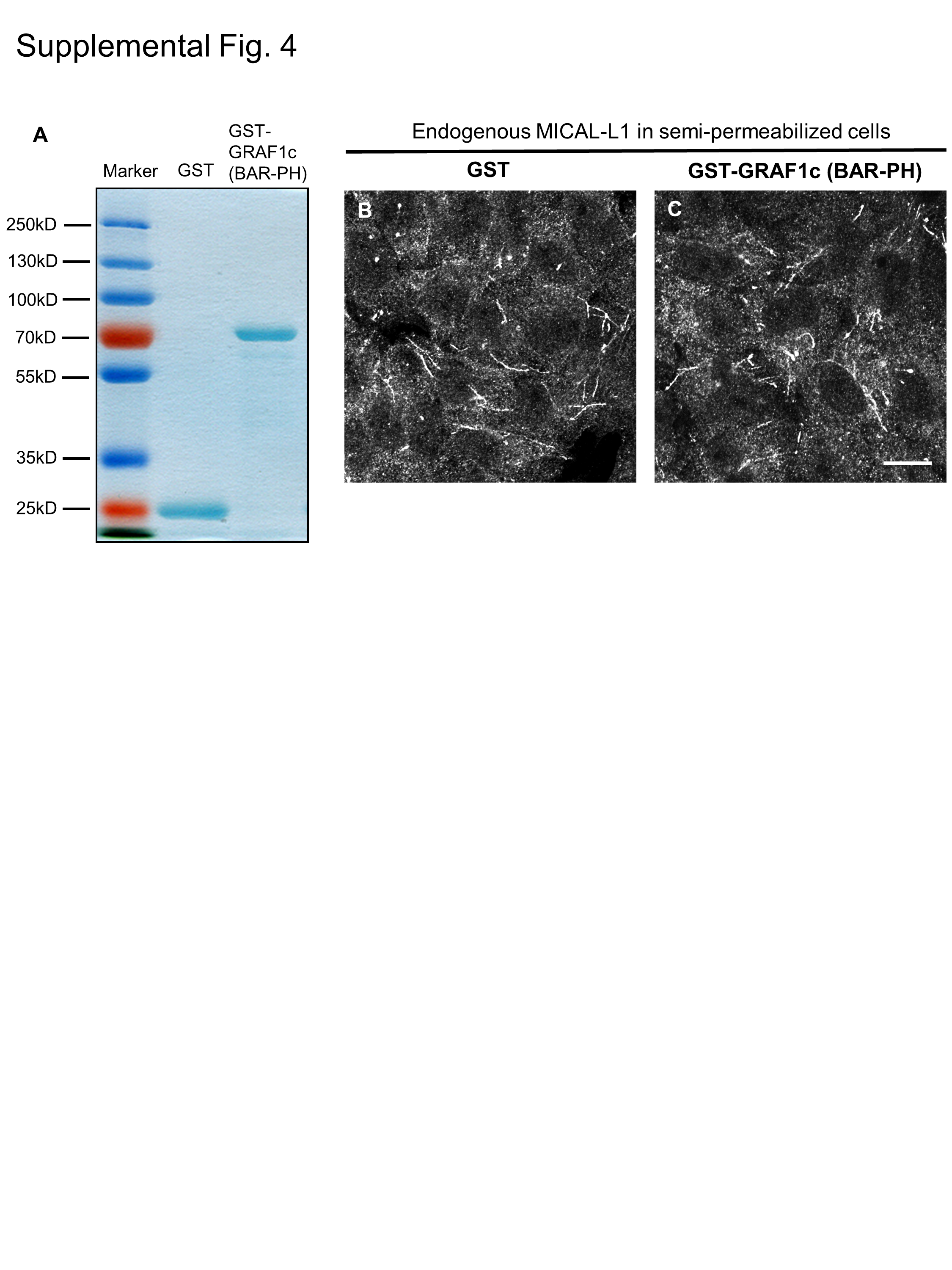

Supplement: Figure S1 — GRAF1c does not interact with MICAL-L1, EHD1, and Syndapin2 by selective yeast two hybrid assays. (A) The Saccharomyces cerevisiae yeast strain AH109 was co-transformed with the indicated GAL4-binding domain (GAL4bd) fusion constructs GAL4bd- GRAF1c and GAL4bd-p53 (control), together with the indicated GAL4 transcription activation (GAL4ad) fusion constructs GAL4ad-EHD1, GAL4ad-MICAL-L1 and GAL4ad-SV40 large T-antigen (control). (B) The interaction between GAL4ad-GRAF1c and GAL4bd-Syndapin2 was analyzed. Cotransformants were assayed for their growth on non-selective (+HIS) and selective (-HIS) media. (C) GRAF1-depletion does not alter the cellular expression levels of either MICAL-L1 or EHD1. Mock-treated or GRAF1-siRNA-treated cells were subject to immunoblotting with anti-MICAL-L1, GRAF1 (control), EHD1 and actin (loading control). (D,E) Cells were transfected with HA-GRAF1c. After 48 h, cells were lysed for 1 h in buffer containing 50 mM Tris pH 7.4, 150 mM NaCl, 0.5% Triton X-100 and protease inhibitors. Cell lysates were incubated with mouse anti-MICAL-L1 antibody or rabbit EHD1 antibody overnight. Protein L beads (D) or Rabbit IP Matrix beads (E) were added to the mixture of cell lysate and MICAL-L1 or EHD1 antibody for 3 h at 4°C. Beads were washed in buffer containing 50 mM Tris pH 7.4, 150 mM NaCl, 0.1% Triton. Proteins were eluted by adding SDS loading buffer. Samples were subjected to 8% SDS-PAGE, followed by blotting with anti-HA and either anti-MICAL-L1 or EHD1 antibodies. [file Presentation1.ZIP › 93240_Caplan_Supplemental_Figure_4.TIF]
